# Optimized protocol for double vaccine immunization against classical swine fever and porcine reproductive and respiratory syndrome

**DOI:** 10.1186/s12917-022-03559-z

**Published:** 2023-01-19

**Authors:** Ziyu Liu, Baiqiang Shan, Chao Ni, Shouhua Feng, Wanting Liu, Xiaoli Wang, Hongtao Wu, ZuofengYang, Jinling Liu, Shu Wei, Changde Wu, Lixia Liu, Zeliang Chen

**Affiliations:** 1 Key Laboratory of Livestock Infectious Disease, Ministry of Education, College of Animal Science & Veterinary Medicine, Shenyang Agricultural University, Shenyang, China, No.120, Dongling Road, Shenhe District, 110866 People’s Republic of China; 2 The Preventive Center of Animal Disease of Liaoning Province, Liaoning Agricultural Development Service Center, No.95, Renhe Road, Shenbei District, Shenyang, 110164 People’s Republic of China

**Keywords:** Classical swine fever, Porcine reproductive and respiratory syndrome, Vaccine, Humoral immunity, Cellular immunity

## Abstract

**Background:**

Classical swine fever and porcine reproductive and respiratory syndrome have seriously affected the development of the swine breeding industry in China. Vaccine immunization remains the main way to prevent these infections. The aim of this study was to establish an optimized protocol for vaccine immunization against classical swine fever virus (CSFV) and porcine reproductive and respiratory syndrome virus (PRRSV).

**Methods:**

Blood samples were collected from the anterior vena cava of pigs after immunization, and blood indices, secreted levels of specific antibodies and neutralizing antibodies associated with humoral immunity, the proliferation capacity of T lymphocytes as a measure of cellular immunity, and secreted levels of IFN-γ and TNF-α were determined.

**Results:**

The results showed that simultaneous immunization against CSFV and PRRSV infections induced strong and specific humoral and T-cellular immune responses, high levels of cytokine IFN-γ secretion and delayed secretion of cytokine TNF-α. Moreover, significantly higher lymphocyte percentages and red blood cell and leukocyte counts were found in the group simultaneously immunized against CSFV and PRRSV. However, no statistically significant differences were observed in hemoglobin values, neutrophil counts, and median cell percentages among the S + PRRS, PRRS-S, and S-PRRS groups.

**Conclusion:**

This study demonstrated that simultaneous immunization against CSFV and PRRSV had the advantages of inducing a rapid, enhanced, and long-lasting immune response. These findings provide a theoretical basis for the establishment of a reasonable and optimized vaccine immunization protocol against CSFV and PRRSV in combination with a variety of other vaccine inoculations.

**Supplementary Information:**

The online version contains supplementary material available at 10.1186/s12917-022-03559-z.

## Background

Classical swine fever (CSF), one of the most important viral diseases in domestic pigs and wild boars, is notifiable to the World Organization for Animal Health (WOAH) [1, 2]. CSF is caused by an enveloped, single-stranded RNA virus belonging to the genus *Pestivirus* in the family *Flaviviridae* [3]. CSF virus (CSFV) infection varies from acute or subacute to chronic forms, depending on the virulence of virus variants. CSF causes tremendous economic losses to the pig industry [4, 5]. Therefore, certain binding legal frameworks exist for CSF surveillance and control in most countries. Of these, emergency and routine vaccinations are the most effective strategies for preventing and controlling CSF in endemic areas. Although CSFV vaccines can induce high levels of immune protection against CSFV infection, immune failures often happen in the field [6, 7].

Porcine reproductive and respiratory syndrome (PRRS) is a swine disease that is characterized by reproductive disorders and respiratory diseases and has a significant economic impact on the swine industry [8, 9]. The etiological pathogenic agent of PRRS is the PRRS virus (PRRSV) [10, 11], a positive-stranded RNA virus that is classified within the family *Arteriviridae* of the order *Nidovirales* [12, 13]. PRRS emerged almost simultaneously in North America and Europe in the late 1980s and then spread quickly to other parts of the world within a few years [14–16]. In June 2006, an unparalleled, large-scale, atypical PRRS outbreak occurred in China, and this disease continues to be a serious problem today. Several studies have confirmed that a highly pathogenic PRRSV is responsible for this disease [17, 18]. Furthermore, the error-prone nature of PRRSV RNA polymerase and its frequent recombination, in addition to the selective pressures in the field, have spurred rapid viral evolution [19–21]. According to current research, vaccination is still considered the key to preventing and controlling PRRS. Therefore, attenuated virulent and inactivated PRRSV vaccines, which are naturally derived from the American and European genotypes, are widely accepted by the pig industry and have been used for decades, however, they fail to provide full protection against various strains [22, 23].

Interestingly, it has been observed that infection with PRRSV prior to vaccination against CSFV causes significant decreases in the antibody response against the CSFV C-strain. 3 weeks after vaccination, serum CSFV C-strain antibody levels were found to be much lower in the group previously infected with PRRSV than in the group that was not [10]. It is well known that PRRSV is an immunosuppressive pathogen that has a serious impact on the immune system, leading to lower host immune function and to secondary infections [24]. In China, an attenuated live CSFV C-strain rabbit-source vaccine and MLV strain PRRS vaccines are widely used for the control of CSF and PRRS, respectively, in domestic pigs. However, these vaccines are not always efficacious in preventing PRRSV and CSFV infections and transmission [25, 26]. An important matter to be addressed is whether prior immunization with a PRRS vaccine has an impact on subsequent CSF vaccination. Thus, it is worthwhile to investigate the effects of PRRS vaccination on the host immune response to CSF vaccination and vice versa, and to select an optimized immunization protocol for CSF and PRRS vaccines. Further, developing a set of relatively complete, scientific, and effective immunization programs for the swine industry would be of great value. Thus, the aim of this study was to establish an optimized vaccine immunization protocol against CSF and PRRS.

## Methods

### Animal facility and ethics statements

Relatively clean piglets, free from PRRSV, CSFV, Pseudorabies virus (PRV), and Porcine circovirus2**(**PCV2) infections, as confirmed by serology tests and virus nucleic acid detection, were housed in a sterilized animal facility. The experimental and animal handling protocols were approved by the Ethics Committee on Experimental Animal Usage and Animal Welfare, Shenyang Agricultural University. A live attenuated PRRS vaccine (**Ingelvac PRRS® MLV,** batch number 245-f85b) was purchased from Boehringer Ingelheim Animal Health Co., Ltd. (USA). A CSF vaccine (live CSFV rabbit-source vaccine, batch number 20200916) was purchased from Shanghai Haili Biotechnology Co., Ltd. (Shanghai, China).

### Experimental groups

In total, 42 healthy weaned Hebao piglets at 7 weeks of age were randomly divided into four groups. Each pig in the S-PRRS group (*n* = 12) was first inoculated intramuscularly with 2 mL of the CSFV vaccine, containing 0.01 g of spleen tissue CSFV, and 7 days later with 2 mL of the PRRSV vaccine at a concentration of 10^4.8^ TCID_50_/mL. Each pig in the PRRS-S group (*n* = 12) was first immunized with the PRRSV vaccine (2 mL, 10^4.8^ TCID_50_/mL) and 7 days later with the CSFV vaccine (2 mL, 0.01 g of spleen tissue CSFV). The S + PRRS group consisted of 12 pigs that were simultaneously immunized with both vaccines, against CSFV and PRRSV, administered at different sites. Six control pigs were kept separately from the vaccinated pigs. The immunization doses were administered in strict accordance with the manufacturers’ instructions. The temperature and clinical signs (anorexia, depression, fever, purple skin discoloration, staggering gait, diarrhea, and cough) were monitored in pigs daily. Blood samples were collected from the anterior vena cava at 0, 10, 20, 30, 40, 50, 60, and 70 days after immunization, and blood indices, secreted levels of specific antibodies associated with humoral immunity, the proliferation capacity of T lymphocytes associated with cellular immunity, and secreted levels of IFN-γ and TNF-α were analyzed.

### Blood index analysis

For routine blood analysis, sera were obtained from the collected blood samples to analyze changes in the numbers of various blood cell types related to immune function using an automated hematology analyzer (Shengda Yixin Medical Technology Co., Ltd., Chengdu, China).

### Humoral immunity tests

The serum antibody levels against CSFV and PRRSV were measured at 0, 10, 20, 30, 40, 50, 60, and 70 days after the vaccinations using a CSFV-specific antibody blocking ELISA kit (IDEXX Biotechnology Co., Ltd., USA), with a blocking rate of the test sample of ≥40% considered positive, and the PRRSV HerdChek® ELISA 3XR kit (IDEXX Biotechnology Co., Ltd.), with a test sample optical density at 650 nm (OD_650_)/positive control OD_650_ (S/P) ratio of > 0.4 considered positive. The procedures described by the manufacturer were utilized for the ELISAs. Humoral immunity characteristics were analyzed based on the detected antibody levels.

### Virus neutralization assay

Virus neutralization assays were performed using MARC-145 cells and PK-15 cells (laboratory preserved), respectively based on the abilities of PRRSV-neutralizing and CSFV-neutralizing antibodies in serum samples to block infection as previously described. Briefly, the sera were heat-inactivated at 56 °C for 30 min, then serum samples were diluted in RPMI1640 medium to create twofold serial dilutions. Each dilution mixed with PRRSV (MOI = 0.1) and CSFV at 0.1 MOI, respectively. Next, the serum-virus mixtures were pre-incubated for 1 hour at 37 °C before they were used for inoculation of MARC-145 cells and PK-15 cells. After inoculation of monolayers in 96-well plates with the mixture and incubation for 1 h, unbound PRRSV and CSFV virions were removed by washing cells with fresh medium. The cells were then incubated for 24 h at 37 °C for further analysis to determine PRRSV and CSFV replication. The maximum dilution titers of the serum samples that reduced PRRSV or CSFV replication by 50% or more were counted as the virus neutralization titers.

### Cellular immunity tests based on transformation T lymphocyte proliferation

Peripheral blood lymphocytes (PBMC) were isolated from the blood of piglets in each group using lymphocyte separation medium (TBD, Tianjin, China) following the manufacturer’s instructions. Briefly, the blood was diluted 1:1 with phosphate buffer saline (PBS) and layered onto lymphocyte separation liquid and then centrifuged at 500×g for 30 min. The PBMC containing interface were subsequently transferred to new tubes and washed three times with Hank’s buffer (pH 7.2). T lymphocytes in PBMC are further isolated with magnetic beads of anti-CD3 antibody, as indicated in the instructions (Meitianni CD3 beans kit,130–050-101, German), and their concentration was adjusted to 2 × 10^7^ cells/mL. Then, the cells were stimulated with one of two treatments: PRRSV (MOI = 0.1) + CSFV (MOI = 0.1) or 5 μL of concanavalin A (ConA), a nonspecific stimulant, each treatment was added to 1 mL of the lymphocyte suspension, and the stimulation index (SI) was analyzed. The SI, also known as the lymphocyte conversion rate, was determined by the MTT method as follows: SI = average OD_570_ value of stimulated cells/average OD_570_ value of unstimulated cells.

### Measurements of IFN-γ and TNF-α concentrations

The serum was obtained at 0, 10, 20, 30, 40, 50, 60, and 70 days after the vaccinations. Subsequently, the concentrations of IFN-γ and TNF-α in the serum were quantitatively determined using a biotin double-antibody sandwich ELISA kit (Dingguo Biotechnology Co., Ltd., Beijing, China). All the operations were performed according to the procedures described for the cytokine ELISA Kits, and the secreted levels of nonspecific immune response factors were assessed.

### Data analysis

Statistical analysis of the data between treatment and control groups was assessed using the GraphPad Prism 6 software (GraphPad Software, La Jolla, CA, USA) Effects of different immunization protocols against CSFV and PRRSV at different time and the 95% confidence intervals were calculated by Probit analysis. All the data were checked for variance homogeneity by Levene’s test and for distribution normality by Shapiro-Wilk’s test. Significant differences were assessed by two-way ANOVA, followed by Tukey’s multiple comparison test. The results are presented as the mean ± standard deviation (SD). *P* < 0.05 was considered significant.

## Results

### Blood index differences among pigs immunized using different vaccination strategies

**Fig. 1:**
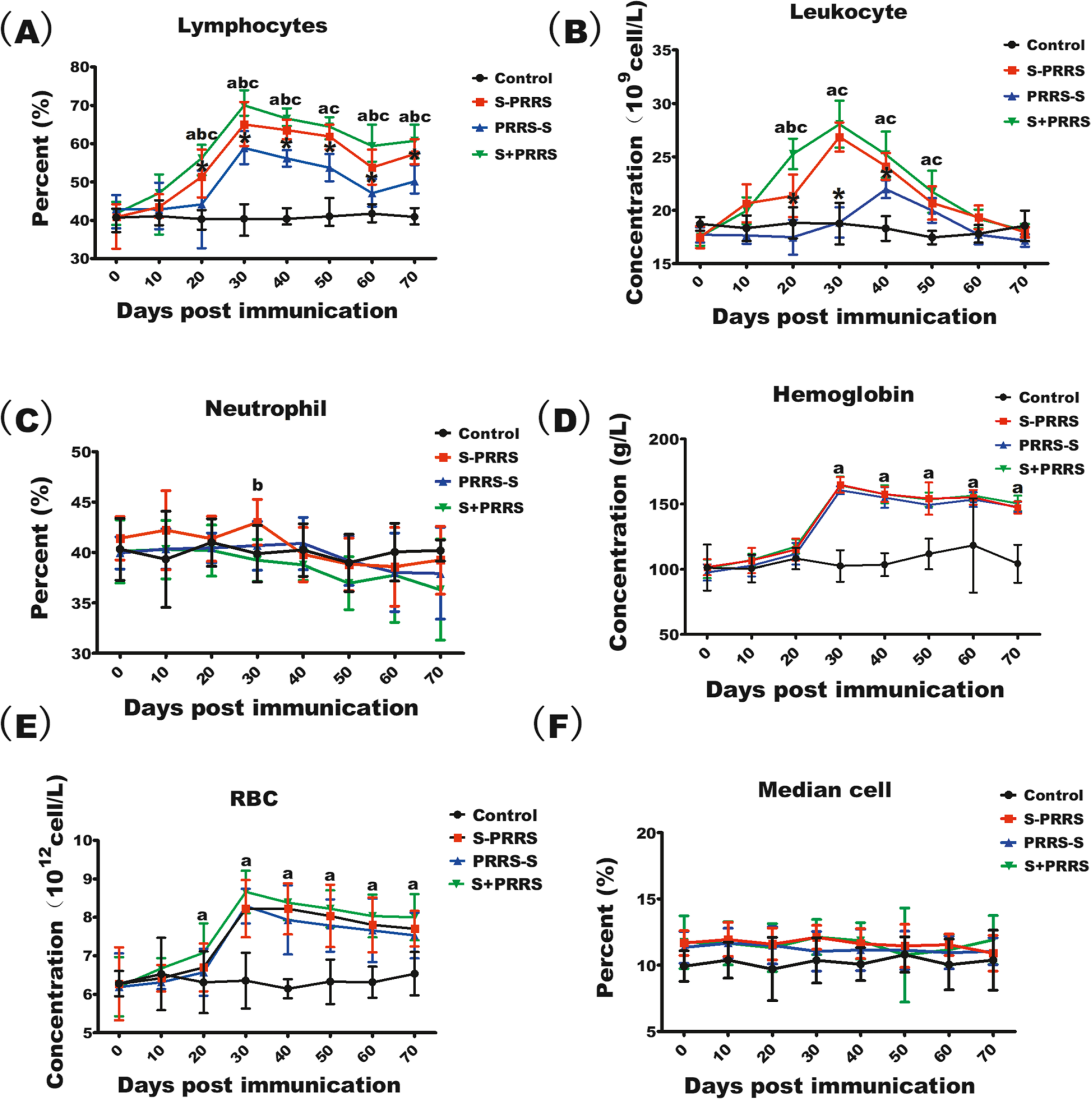
Effects of different immunization schedules against CSFV and PRRSV on swine blood indices. **A** Mean lymphocyte proportions in different treatment groups. **B** Mean leukocyte counts in different treatment groups. **C** Mean neutrophil proportions in different treatment groups. **D** Mean hemoglobin concentrations in different treatment groups. **E** Mean red blood cell numbers in different treatment groups. **F** Mean median cell ratios in different treatment groups. Note: the bars are the respective standard deviation. Data are shown as the mean ± SD. Values with small letter (**a**) represent significant differences between S + PRRS group and control group within different immunization time (*P* < 0.05); Values with small letter (**b**) represent significant differences between S + PRRS group and S-PRRS group within different immunization time (*P* < 0.05); Values with small letter (**c**) represent significant differences between S + PRRS group and PRRS-S group within different immunization time (*P* < 0.05);Values with (*) represent significant differences between S-PRRS group and PRRS-S group (*P* < 0.05);(**ns**) indicates no significant differences among S + PRRS,S-PRRS, PRRS-S and control groups within different immunization time (*P* > 0.1)

The results of statistical analysis of blood indices are shown in Fig. 1. The mean lymphocyte proportions in the S + PRRS, PRRS-S and S-PRRS groups showed upward trends after immunization, reaching a peak at approximately 30 days (Fig. 1A). The mean peak lymphocyte proportion in the S + PRRS group was significantly higher than those in the PRRS-S and S-PRRS groups (*P* < 0.05), and after day 30, this value slowly declined and finally leveled off. However, it was still significantly higher than those observed for the PRRS-S and S-PRRS groups (P < 0.05, whereas there was significant difference in the mean lymphocyte proportions between the PRRS-S and S-PRRS groups (*P* < 0.05).

As shown in Fig. 1B, unlike those in the control group, the mean leukocyte counts in the S + PRRS an S-PRRS groups showed upward trends after immunization and reached their peak values on day 30. Moreover, the mean leukocyte counts were significantly higher in the S + PRRS and S-PRRS groups than in the PRRS-S group (*P* < 0.05). Although S + PRRS and S-PRRS groups are not significantly different on days 40–50 (*P* > 0. 1). Whereas that in the PRRS-S group reached its peak value later (on day 40). Thereafter, the mean leukocyte counts in the three groups (S + PRRS, PRRS-S, and S-PRRS) gradually declined and finally stabilized, without significant differences among them on days 60–70 (*P* > 0. 1).

Overall, the mean neutrophil proportions in the S + PRRS, PRRS-S, S-PRRS, and control groups tended to be stable, with no significant difference before and after immunization in each group (*P* > 0.1). Furthermore, the mean neutrophil proportion of S + PRRS group was lower that of S-PRRS group on the 30th day (*P* < 0.05), no significant differences were observed among the groups (*P* > 0. 1) (Fig. 1C).

The mean hemoglobin concentrations in the S + PRRS, PRRS-S, and S-PRRS groups showed overall upward trends, reaching peak values 30 days after immunization, and then tended to be stable, with no significant differences among the three immunization groups (*P* > 0.1), however, there were significant differences when compared with the control group (*P* < 0.01) (Fig. 1D).

As shown in Fig. 1E, the mean red blood cell numbers in the S + PRRS, PRRS-S, and S-PRRS groups showed overall upward trends. All of them significantly increased and reached peak values approximately 30 days after immunization and then tended to be stable during days 40–70, with no significant differences among the three immunization groups (*P* > 0. 1). However, there was significant difference of S + PRRS group when compared with the control group (*P* < 0.05).

The mean median cell ratios in the S + PRRS, PRRS-S, S-PRRS, and control groups were stable, with no significant differences among the groups (*P* > 0. 1) (Fig. 1F).

### Effects of the different immunization schedules on humoral immunity

**Fig. 2:**
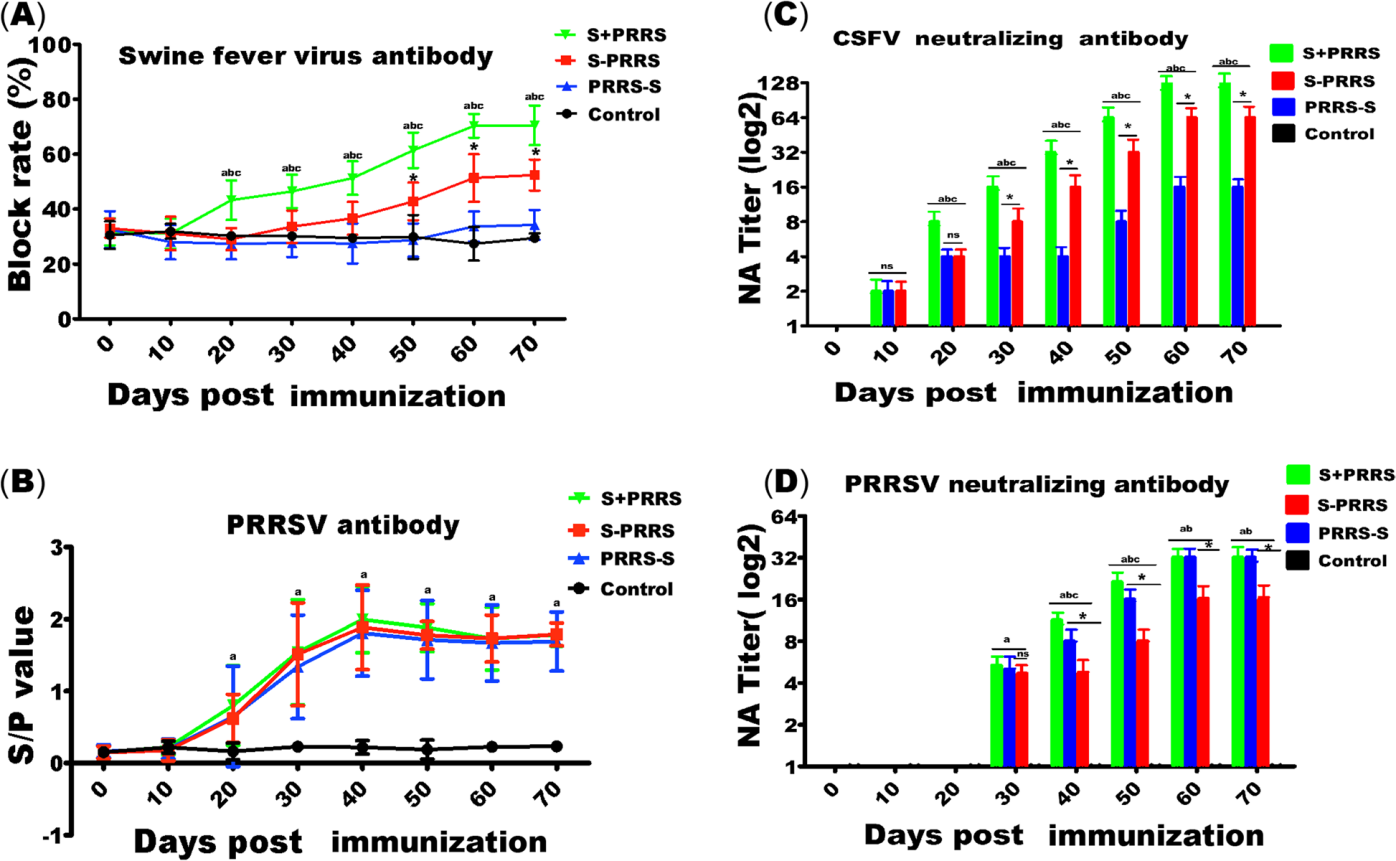
Serum levels of PRRSV- and CSFV-specific antibodies in different treatment groups. **A** Serum antibody levels against CSFV. The antibody blocking rate of a test sample greater than or equal to 40% was considered positive. **B** Serum antibody levels against PRRSV. S/P ratios of greater than 0.4 were considered positive. **C** The titer of serum neutralizing antibody against CSFV. **D** The titer of serum neutralizing antibody against PRRSV. Note: the bars are the respective standard deviation. Data are shown as the mean ± SD, and different letters **(a, b, c)** and (*) above the bars indicate significant differences (*P* < 0.05) as in Fig. 1; **(ns)** indicates no significant differences among S + PRRS, S-PRRS, PRRS-S and control groups within different immunization time (*P* > 0.1)

The final results of statistical analysis of the serum antibody levels against CSFV are shown in Fig. 2A. In all groups, the average CSFV antibody blocking rates did not change for approximately 10 days after immunization but started to increase in the S + PRRS and S-PRRS groups at approximately 20 and 30 days after immunization, respectively. The average blocking rates in the S + PRRS group were significantly higher than those in the PRRS-S (*P* < 0.05), S-PRRS (*P* < 0.05) and control groups (*P* < 0.05) between approximately 20 and 70 days after immunization, whereas the lowest value was observed in the PRRS-S group on day 50. The differences in the antibody blocking rates on days 50–70 were significant (*P* < 0.05) in the S + PRRS and S-PRRS groups but not in the PRRS-S group before and after immunization.

The PRRSV serum antibody levels in the S + PRRS, PRRS-S, and S-PRRS groups increased compared with those in the control group based on the average S/P ratios (Fig. 2B). Significant S/P ratio increases were observed approximately 20 days after immunization, and peaks were reached on approximately day 40 after immunization, after which the levels started to plateau. The differences in the antibody levels before and after immunization were significant in the S + PRRS, PRRS-S, and S-PRRS groups (*P* < 0.05), but no significant differences were observed among the groups (*P* > 0. 1) (Fig. 2B).

### Neutralization antibody titers based on different immunization protocols

Overall neutralizing antibody (NA) titers with various pig sera are summarized in Fig. 2C-D. The NA titers against CSFV in the S + PRRS group (1:2, 1:8, 1:16, 1:32,1:64 and 1:128) were higher than those against S-PRRS group (1:2, 1:4, 1:8, 1:16,1:32 and 1:64) (*P* < 0.05) and PRRS-S group (1:2, 1:4, 1:8, and1:16) (*P* < 0.05). Meanwhile, the S-PRRS group were higher compared to PRRS-S group against CSFV. The NA titers against PRRSV in the S + PRRS group (1:6, 1:8, 1:16 and 1:32) were higher than those against S-PRRS group (1:6, 1:8, and1:16) on days 40–70 (*P* < 0.05), and PRRS-S group (1:6, 1:8, 1:16 and 1:32) on day on days 40–50 (*P* < 0.05), and almost equivalent to PRRS-S group on day 60–70. Meanwhile, the PRRS-S group were higher comparable to S-PRRS group against PRRSV on days 40–70 (*P* < 0.05).

In addition, the S + PRRS group was almost equivalent to PRRS-S and S-PRRS groups on day 30. Almost no neutralizing antibodies against PRRSV were produced during the first 30 days after immunization for the three groups.

### Impacts of the different immunization protocols on T lymphocyte proliferations

**Fig. 3:**
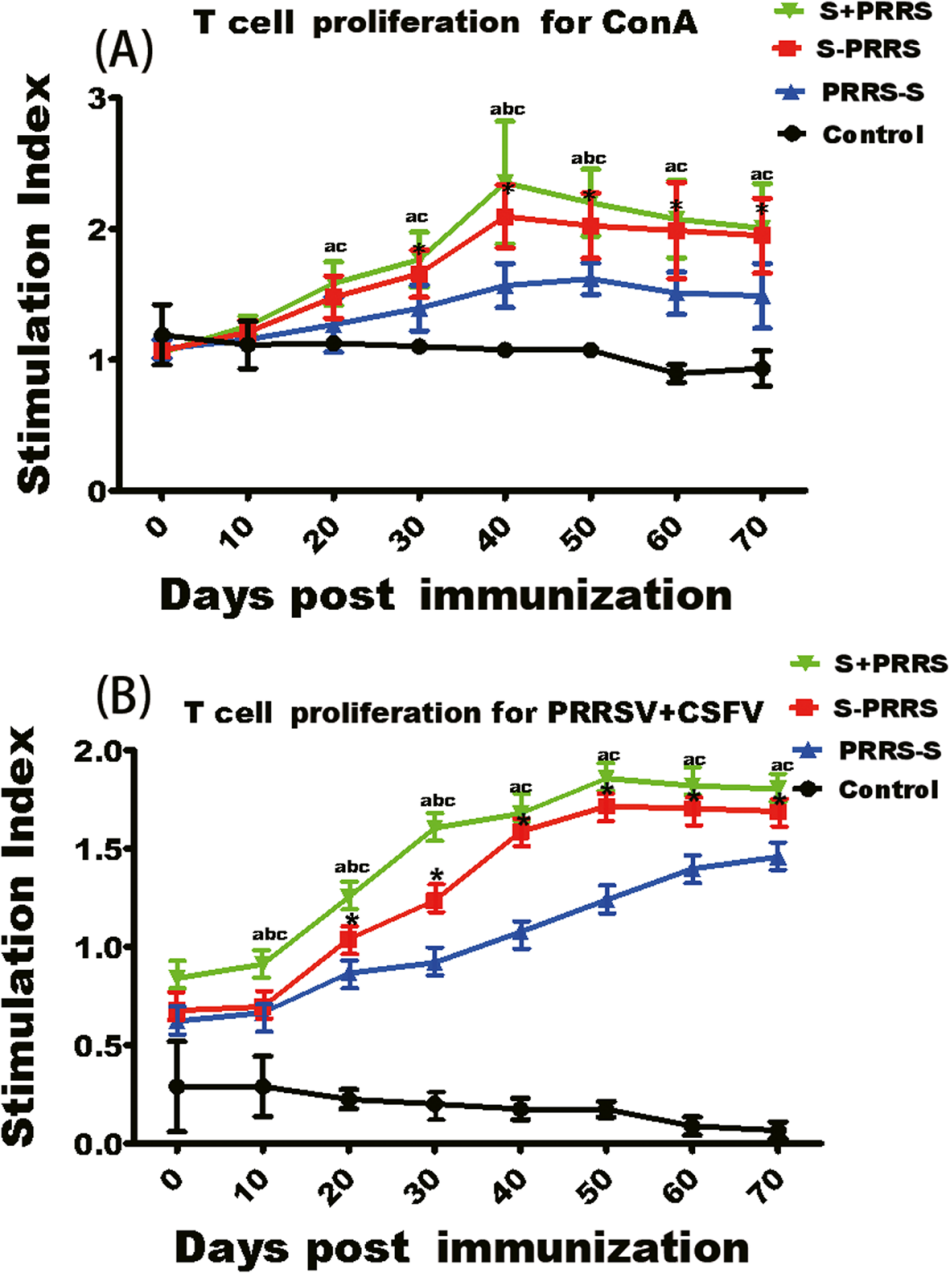
Effects of different immunization protocols against CSF and PRRS on swine cellular immunity. **(A)** Stimulation indices of T lymphocytes at the indicated time points after vaccinations. The T-lymphocyte conversion rate for ConA was determined based on the stimulation index. **(B)** The T-lymphocyte conversion rate for CSFV+PRRSV was determined based on the stimulation index. Note: the bars are the respective standard deviation. Data are shown as the mean ± SD, and different letters **(a, b, c)** and (*) above the bars indicate significant differences (*P* < 0.05) as in Fig. 1; **(ns)** indicates no significant differences among S + PRRS, S-PRRS, PRRS-S and control groups within different immunization time (*P* > 0.1)

The final results of statistical analysis of the SI data are shown in Fig. 3A. The average SI in the S + PRRS group reached a peak at approximately 40 days after immunization and was significantly higher than those in the S-PRRS, PRRS-S and control group on days 40–50 (*P* < 0.05), not obviously different that in the S-PRRS group on days 20–30 and 60–70 (*P* > 0.1). In addition, there were differences between PRRS-S and S-PRRS groups on days 30–70 (*P* < 0.05). As shown in Fig. 3B, the SIs of S + PRRS, PRRS-S, and S-PRRS groups showed an overall upward trend. The increase in virus specific SI of T lymphocytes, stimulated with the PRRSV and CSFV vaccines, was significant in the S + PRRS group on days 10–30 post the immunization compared with S-PRRS, PRRS-S and control groups (*P* < 0.05), and significant on days 40–70 compared with PRRS-S group (P < 0.05), but the levels in the S + PRRS group has no difference to that of the S-PRRS group (*P* > 0.1). These results indicate the PRRSV and CSFV vaccines could effectively stimulate a specific cellular immune response which was strongest when inoculated simultaneously.

### Differences in the secreted concentrations of IFN-γ and TNF-α depending on the immunization protocol

**Fig. 4:**
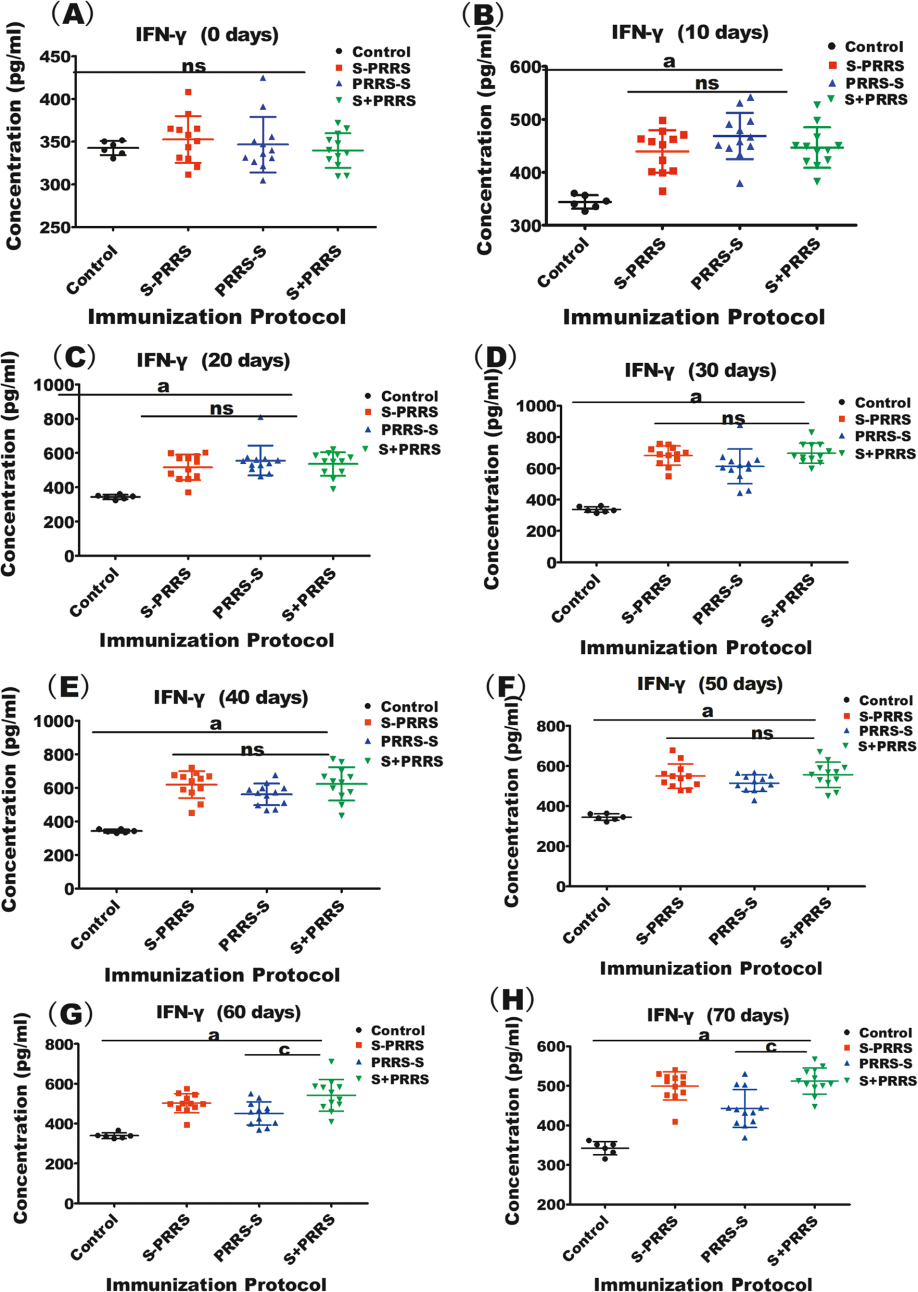
Effects of different immunization protocols against CSF and PRRS on the concentration of IFN-γ in swine. (A–H) Concentrations of IFN-γ at the indicated time points after vaccinations. Note: the bars are the respective standard deviation. Data are shown as the mean ± SD, and different letters **(a, b, c)** and (*) above the bars indicate significant differences (*P* < 0.05) as in Fig. 1; **(ns)** indicates no significant differences among S + PRRS, S-PRRS, PRRS-S and control groups within different immunization time (*P* > 0.1)

The mean serum IFN-γ concentrations were not significantly different among the three immunization groups but were significantly higher than those in the control group from day 10 post-vaccination (*P* < 0.05). The mean IFN-γ levels were higher in the S + PRRS group than in the PRRS-S group on days 60 and 70 after vaccination (both *P* < 0.05) (Fig. 4).

**Fig. 5:**
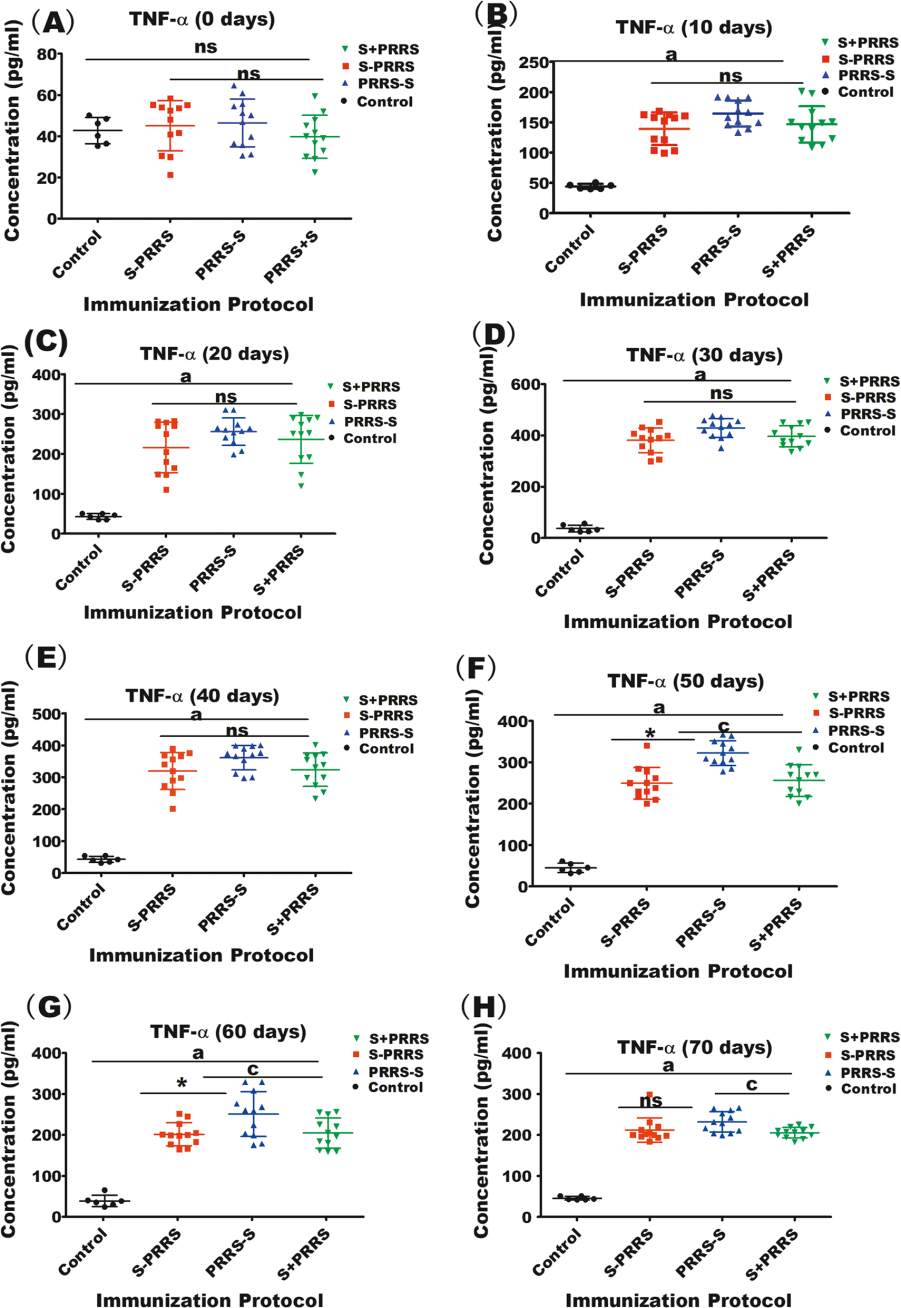
Effects of different immunization protocols against CSF and PRRS on the concentration of TNF-α in swine. (A–H) Concentrations of TNF-α at the indicated time points after vaccinations. Note: the bars are the respective standard deviation. Data are shown as the mean ± SD, and different letters **(a, b, c)** and (*) above the bars indicate significant differences (*P* < 0.05) as in Fig. 1; **(ns)** indicates no significant differences among S + PRRS, S-PRRS, PRRS-S and control groups within different immunization time (*P* > 0.1)

Similarly, from day 0–70 post vaccination, the level of TNF-α in the pigs inoculated with PRRSV and CSFV vaccines simultaneously was higher than in the control group (*P* < 0.05). Which can promote antigen presentation to helper T lymphocytes. However, TNF-α was lower in the S + PRRS group than in the PRRS-S group on days 50–70 (*p* < 0.05), while the difference between the PRRS-S and S-PRRS pigs was not significant from day 0–70 post vaccination (*p* > 0.1) except for day 50–60 (Fig. 5).

## Discussion

The CSFV C-strain vaccine is regarded as one of the most effective CSFV vaccines. However, immune failures of CSFV vaccines are frequently encountered for many reasons [27]. Previous research has demonstrated that the PRRSV vaccine also induced delayed neutralizing antibody and cell-mediated immune responses in pigs, similar to natural PRRSV infection [28, 29]. PRRS is a serious infectious disease that can cause immunosuppression. As failed immunization protocols bring great economic losses to the swine industry, care should be taken during vaccine selection and the development of an immunization program.

In this study, we investigated the antibody responses, blood indices, proliferation capacity of T lymphocytes associated with cellular immunity, and secreted levels of IFN-γ following vaccinations against CSFV and PRRSV using different immunization strategies. Swine simultaneously immunized with the vaccines against CSFV and PRRSV demonstrated a rapid, enhanced, and long-lasting immune response. The antibody response, cellular response, and percentages of lymphocytes, leukocytes, and red blood cells in the S + PRRS group were much higher than those in the other groups. By contrast, IFN-γ secretion and T-lymphocyte proliferation were lower, and the antibody level against CSFV was the lowest, meanwhile TNF-α secretion was the highest in the group immunized first with the PRRSV vaccine and then with the CSFV vaccine. In view of this research result, related research clarified that PRRSV induced an elevated level of a subset of pro-inflammatory cytokines, especially TNF-α, through the nuclear factor κB (NF-κB) signaling pathway to inhibit the replication of CSFV-C in vitro. Thus, PRRSV-induced CSFV vaccination failure, and this has an important implication in CSF vaccination and control [30–32].

The potential role of PRRSV in regulating the host immune system is supported by the fact that the virus preferentially replicates and persists in macrophages, which implies that PRRSV has the ability to escape from or modulate the host immune system [13]. Once it invades the pig body, PRRSV first attacks macrophages in the lungs and can severely damage the phagocytic, bactericidal, and secretory functions of macrophages, resulting in decreased immune function [33]. Macrophages, which participate in both nonspecific and specific immune responses, are a very important immune cell type and an important member of the body’s immune system [34]. Macrophages play an important “scavenger” role in the early stage of a viral infection. In addition, they are capable of antigen presentation and after being activated by antigen stimulation, secrete cytokines, which can promote antigen presentation by helper T lymphocytes and the transformation of B lymphocytes into plasma cells to secrete immunoglobulin to fight against and eliminate the invading pathogen [12]. Therefore, it is presumed that after the injection of an attenuated PRRSV vaccine, a certain amount of the attenuated PRRSV might affect macrophage antigen processing and presentation, resulting in the inability of B lymphocytes to proliferate in large quantities and convert into plasma cells to secrete antibodies. This also leads to low levels of the proliferation and transformation of T lymphocytes and reduces the ability of macrophages to activate T cells and secrete cytokines, such as IFN-γ. The reduction in IFN-γ levels also affects humoral immunity because IFN-γ can directly act on B lymphocytes to promote their transformation [35]. If the CSF vaccine is injected at this time, the swine body, with its impaired immune function and humoral immunity, is not able to produce a large amount of CSFV antibodies; this causes interference with the establishment of immunity to the CSFV vaccine and may even lead to immune failure [26].

Additionally, some studies have reported the kinetics of proinflammatory cytokine responses, such as those of IL-10, IFN-α, and TNF-α, following PRRSV infection [25, 26]. In these studies, significant upregulation of IL-10 expression and downregulation of IFN-γ expression were observed when PBMCs from animals previously immunized with a CSFV vaccine were treated with a recall antigen in the presence of PRRSV [36, 37].

This research further illustrated that the viruses evade host immunity by promoting the secretion of IL-10, TNF-α, and TGF-β, which antagonize the protective Th1 immune response [37, 38]. Nonetheless, the expression of both IL-10- and TGF-β-encoding genes was increased in pigs vaccinated against PRRSV by a systemic route. Apart from this, infiltration of Tregs into the infected pig lungs contributes to the secretion of high levels of IL-10 and TGF-β. This important T-cell subset was associated with the protection in pigs vaccinated against PRRSV [10], which might have major consequences for the immunosuppressive effects of PRRSV on the host immune response.

On the other hand, this study and some related studies have demonstrated that the specific antibody immune response to PRRSV is not apparent until 7–10 days after infection, whereas neutralizing antibodies do not appear until 4 weeks after infection [39]. Notably, specific T cells can be detected in swine blood 7 or 14 days after infection with PRRSV. Thus, CD4^+^ T cells play a crucial role in the specific immune response against PRRSV infection [40]. Furthermore, the key cytokine associated with a host cell-mediated immune response is IFN-γ, which is produced by NK cells, γδT cells, Th cells, CTLs, and Th memory cells. The level of IFN-γ in the serum is a nonspecific indicator that can reflect the overall cellular immunity level of the body at a certain stage [41]. Therefore, cellular immunity plays a very important role in the immune response in the absence of neutralizing antibodies when the process of swine PRRSV infection in the animal body is detected. However, both live and inactivated PRRSV could significantly affect cell-mediated and humoral immunity induced by the CSF vaccine [25].

The results of the present study may provide new evidence for the establishment of a reasonable and optimized vaccine immunization program against CSF and PRRS in combination with a variety of other vaccine inoculations. It should also be considered that the discrepancies in the results of similar reports might have resulted from differences in the strains of PRRSV, secondary pathogens, age of piglets, and immunization schedules used in different studies [10]. We preliminarily believe that vaccines from different strains could have different immunization effects. The specific mechanism of vaccine immunity will be investigated in our subsequent study to further enrich and improve immune prevention of PRRSV and CSFV infections.

## Conclusions

Our findings provide an initial assessment of the effects of different immunization protocols against CSFV and PRRSV on the host immune system. This study demonstrated that simultaneous immunization against CSFV and PRRSV had the advantages of inducing a rapid, enhanced, and long-lasting immune response. These findings provide a theoretical basis for the establishment of a reasonable and optimized vaccine immunization program against CSFV and PRRSV in combination with a variety of other vaccine inoculations.

## Supplementary Information

**Additional file 1:** **Table S1.** The raw data on the detection of different immunization protocols for CSFV and PRRSV vaccines at different time periods before and after immunization.

**Additional file 2:** **Table S2–1.** The analysis data on the immune cell of different immunization protocols for CSFV and PRRSV vaccines at different time periods before and after immunization based on raw data.

## Data Availability

All data generated and/or analyzed during this study are included in this manuscript. The raw data are available from the corresponding author on reasonable request or referring to supplementary materials.

## Abbreviations

CSFV: Classical swine fever virus
PRRSV: Porcine reproductive and respiratory syndrome virus
WOAH: World Organization for Animal Health
Con A: Concanavalin A
SI: Stimulation index
