# Correction: Optimized protocol for double vaccine immunization against classical swine fever and porcine reproductive and respiratory syndrome

**DOI:** 10.1186/s12917-023-03595-3

**Published:** 2023-02-09

**Authors:** Ziyu Liu, Baiqiang Shan, Chao Ni, Shouhua Feng, Wanting Liu, Xiaoli Wang, Hongtao Wu, ZuofengYang, Jinling Liu, Shu Wei, Changde Wu, Lixia Liu, Zeliang Chen

**Affiliations:** 1 Key Laboratory of Livestock Infectious Disease, Ministry of Education, College of Animal Science & Veterinary Medicine, Shenyang Agricultural University, Shenyang, China, No.120, Dongling Road, Shenhe District, 110866 People’s Republic of China; 2 The Preventive Center of Animal Disease of Liaoning Province, Liaoning Agricultural Development Service Center, No.95, Renhe Road, Shenbei District, Shenyang, 110164 People’s Republic of China

**Correction: BMC Vet Res 19, 14 (2023)**

**https://doi.org/10.1186/s12917-022-03559-z**

In the originally published version of this article [1], the names of the authors (Shu Wei, Changde Wu, Lixia Liu) were mistakenly included in the consent form. The original article has been corrected.
